# A novel comorbidity index in Italy based on diseases detected by the surveillance system PASSI and the Global Burden of Diseases disability weights

**DOI:** 10.1186/s12963-023-00317-7

**Published:** 2023-10-30

**Authors:** Angela Andreella, Lorenzo Monasta, Stefano Campostrini

**Affiliations:** 1 Department of Economics, Ca’ Foscari University of Venice, Venice, Italy; 2 Institute for Maternal and Child Health, IRCCS Burlo Garofolo, Trieste, Italy

**Keywords:** Health-statistics, Comorbidity, Disability weights, Surveillance system PASSI, Global burden of disease project

## Abstract

**Background:**

Understanding comorbidity and its burden characteristics is essential for policymakers and healthcare providers to allocate resources accordingly. However, several definitions of comorbidity burden can be found in the literature. The main reason for these differences lies in the available information about the analyzed diseases (i.e., the target population studied), how to define the burden of diseases, and how to aggregate the occurrence of the detected health conditions.

**Methods:**

In this manuscript, we focus on data from the Italian surveillance system PASSI, proposing an index of comorbidity burden based on the disability weights from the Global Burden of Disease (GBD) project. We then analyzed the co-presence of ten non-communicable diseases, weighting their burden thanks to the GBD disability weights extracted by a multi-step procedure. The first step selects a set of GBD weights for each disease detected in PASSI using text mining. The second step utilizes an additional variable from PASSI (i.e., the perceived health variable) to associate a single disability weight for each disease detected in PASSI. Finally, the disability weights are combined to form the comorbidity burden index using three approaches common in the literature.

**Results:**

The comorbidity index (i.e., combined disability weights) proposed allows an exploration of the magnitude of the comorbidity burden in several Italian sub-populations characterized by different socioeconomic characteristics. Thanks to that, we noted that the level of comorbidity burden is greater in the sub-population characterized by low educational qualifications and economic difficulties than in the rich sub-population characterized by a high level of education. In addition, we found no substantial differences in terms of predictive values of comorbidity burden adopting different approaches in combining the disability weights (i.e., additive, maximum, and multiplicative approaches), making the Italian comorbidity index proposed quite robust and general.

## Introduction

The term comorbidity indicates the simultaneous presence of two or more diseases in the same person. These conditions can be related or unrelated and co-occur one after the other [1, 2]. Comorbidity is common, especially in older people and those with chronic diseases. It can complicate the diagnosis and treatment of individual conditions and impact the person’s overall health and well-being [3].

Understanding comorbidity is vital for supporting health-related decision-making processes. It helps policymakers, healthcare providers, and other stakeholders identify the most pressing health issues a population faces and allocate resources accordingly [4]. It is well known in the literature that having several chronic diseases impacts people’s lives in several ways, such as the quality of life, psychological difficulties, higher mortality but also longer hospital stays, higher treatment costs, and more postoperative complications. This thus turns out to be a substantial burden on the individual and cost for health systems, as well as a problem from an organizational standpoint for hospitals and health centers [5]. Morbidity and comorbidity data can help researchers and scientists identify trends and patterns in the occurrence of different diseases and conditions, which can inform the development of new treatments and prevention strategies [6, 7]. Analyzing comorbidity is crucial to fully grasp the resulting disabilities and the ensuing burden they bring. Calculating this burden is pivotal to determining the impact on specific population segments and setting public health priorities. In particular, the importance of analyzing morbidity and comorbidity and their burden on subjects’ lives is growing year after year due to the gradual increase in population aging [8]. However, this population analysis is challenging beyond the issues of collecting and analyzing sensitive data such as health data.

The concept of comorbidity burden is complex and multidimensional. For that, several definitions can be found in the literature [9]. Three main reasons can be identified: (i) analyzing comorbidity depends on the type of available data (e.g., the type of detected diseases in the surveillance/study considered, study objective, target population), (ii) the burden of disease on a person’s health depends on the severity or duration of the diseases or conditions, (iii) the presence of multiple morbidities must be aggregated in some way to offer a measure of comorbidity. For example, some studies define comorbidity as the presence of two or more conditions simultaneously in a patient. At the same time, other studies also include complications of an existing condition as comorbidity. In addition, comorbidity can be assessed using specific scales such as the Charlson’s index [10–12] or the Elixhauser one [13, 14], analysis of patient records or the use of administrative data such as health care billing data [15].

Focusing on the Italian population, Corrao and colleagues [16] propose a multi-source comorbidity score using several sources of information from the administrative Italian National Health System (NHS) databases. The comorbidity was then described by an index composed of 34 variables and weights defining the burden of diseases estimated by a Weibull survival model. This index depends entirely on the data available (i.e., administrative NHS databases), not open-source for privacy reasons. Applying this index to other data, such as surveillance health system survey data, is therefore impossible. Some researchers have linked different data sources [17, 18], but it is more the exception than the rule. Focusing, therefore, on the Italian population and also on the data that we have available, we must cite the work of Pastore et al. [19]. They [19] define an index of morbidity as a binary variable describing the presence of at least one disease over ten detected diseases. Pastore and colleagues [19] used data from the Italian Non-Communicable Diseases (NCDs) surveillance system PASSI [20], a monthly cross-sectional study where self-declared health status, diagnosed diseases, risk factors, and sociodemographic variables are recorded. The PASSI data comprise a regional and national representative sample of the population between the ages of 18 and 69 who are residents of Italy, registered within the health registry, not institutionalized (neither hospitalized nor residing in educational or rehabilitation facilities).

Therefore, the index proposed by Pastore et al. [19] is pretty simple. The concept of comorbidity and the burden of disability associated with each disease is not taken into account, as is the burden of having at least one disease versus having no disease. The authors themselves, in fact, suggest using some weights to take into account the level of possible disability coming from each detected disease.

For that, in this work, we propose a new comorbidity burden index, focusing on the Italian framework. We then analyze the same surveillance system data used by Pastore et al. [19] (i.e., PASSI), considering as disease weights the ones coming from the Global Burden of Diseases (GBD) project [21, 22]. These weights, called disability weights, reflect the magnitude of health loss linked with specific health conditions [23]. Disability weight is an important factor in estimating the amount of time lost to health due to living with a particular disease state [24]. The GBD defined the first set of disability weights in 1996 [25]; after that, several alternatives were proposed characterized by different design choices [26]. Please refer to [24, 26] for a complete review. The ones we will consider in this work are computed using data from surveys based on paired comparison questions. Respondents must consider two hypothetical individuals with different names of health states (randomly selected) and indicate which is healthier [23]. Many factors can influence the computation of the disability weights, i.e., the health state description, the panel of judges, the valuation methods for the health states, the time presentation, and the surveying techniques [24]. However, this paper focuses on defining a novel comorbidity burden index rather than novel disability weights. We decided then to use the 2019 GBD disability weights having been tested and validated several times [24, 26]. In the manuscript, when discussing the proposed comorbidity index, we refer to an index that considers the burden of multiple diseases in subjects’ lives. The terms “comorbidity index,” “comorbidity burden index,” and “combined disability weights” are therefore interchangeable throughout the manuscript.

The outline of the paper is as follows. We show the steps to create the comorbidity index based on the GBD disability weights and the diseases declared in the Italian surveillance system PASSI. An example of how using this novel comorbidity index (i.e., random forest [27]) is then subsequently provided. This analysis allows an understanding of the comorbidity level and the associated disability burden in Italian sub-populations characterized by different socioeconomic statuses. Finally, conclusions and further directions are summarized at the end of the manuscript.

## Building the comorbidity burden index

This section outlines the steps in creating the Italian comorbidity index, which permits the analysis of the relationship between the burden of diseases and socioeconomic factors such as age and sex, but also economic and educational statuses, which are rarely available information in hospital records or similar sources. The first subection briefly describes the data used to build this novel Italian comorbidity index, while the second one defines the procedure for computing it.

### Data

We use data from the Italian surveillance system PASSI which collects by sample surveys information on lifestyles and behavioral risk factors related to the occurrence of NCDs focusing on the Italian adult population (i.e., people from 18 to 69 years old). For further information, please see the work of Baldissera et al. [20] and the following web page https://www.epicentro.iss.it/passi/en/english. We focus on 2019 data composed of 31,746 interviews.

In particular, we consider the following questions:

1. “Has a doctor ever diagnosed or confirmed you with one or more of the following diseases?”
2. “How is your overall health?”

joining with sex, age, educational level, and economic problems information. Regarding the first question, the respondents can self-report the following health conditions: diabetes, kidney failure, bronchitis/emphysema/respiratory failure, myocardial infarction/cardiac ischemia/coronary artery disease, tumor (including leukemias and lymphomas), chronic liver disease/cirrhosis, stroke/cerebral ischemia, heart diseases (e.g., valvulopathy decompensation), bronchial asthma, and arthrosis/arthritis (e.g., rheumatoid, arthritis, gout, lupus, fibromyalgia). We can note that the diseases detected in PASSI are the most frequent NCDs at the Italian/European level. Instead, the second question focuses on capturing the perceived health as an ordinal categorical variable that takes values between 1 and 5, where 1 means excellent self-reported health and 5 means very bad self-reported health.

Finally, the educational level has been coded here as a binary variable equal to low if the respondent has an education level below high school and high otherwise. The economic problem variable has also been coded as a binary variable taking value equal to high if the respondent makes ends meet with the financial resources available (from own or family income) very/quite easily and low otherwise. The sample consists of $51.4\%$ women, $56\%$ have economic problems, and $67.8\%$ have a high formal education level. The average age is 45 years, and the variable is uniformly distributed. To better understand the distribution of these sociodemographic variables as a whole, Fig. 6 in Appendix 1 shows the density distribution of the variable age of the PASSI sample for each combination between the levels of the variable sex, educational (low-high), and economic (no economic problems-economic problems) levels. For further details on these socioeconomic variables and PASSI data collection, please refer to [19, 20].

The second data set that we use to construct the comorbidity index is the disability weights coming from the GBD 2019 study [23, 28]. The disability weights describe the magnitude of health loss related to 440 health states, i.e., diseases, injuries, and risk factors estimated across 204 countries [29]. These weights are measured on a scale from 0 to 1, where 0 equals a state of full health, and 1 equals a state of death. The GBD estimates are downloadable from https://ghdx.healthdata.org/record/ihme-data/gbd-2019-disability-weights.

The following subsection shows how the novel comorbidity burden index is defined.

### Method

In the introduction, we mentioned that currently, there is no gold standard method to define an index that describes the magnitude and burden of individual comorbidity. Here, we will focus on a novel definition of a comorbidity burden index based on the data presented in the previous subsection, i.e., the most frequent NCDs in the Italian population.

When the aim is to analyze the comorbidity in a population, one must take into account that the impact on a person’s life of a given disease depends on the severity of this disease. In addition, since the same individual can declare more than one disease, we must define a way to aggregate multiple health conditions to define comorbidity. In order to take these two aspects into account, we use the disability weights coming from the GBD [23]. We must associate each disease measured by the PASSI surveillance system with one weight of disability from GBD. However, in this step, we must deal with several problems. First, the GBD provides weights for 440 diseases, whereas PASSI only examines ten NCDs. Second, for each disease analyzed, the GBD provides different weights depending on the severity of the disease. To solve these two problems, we moved in two steps.

**Fig. 1:**
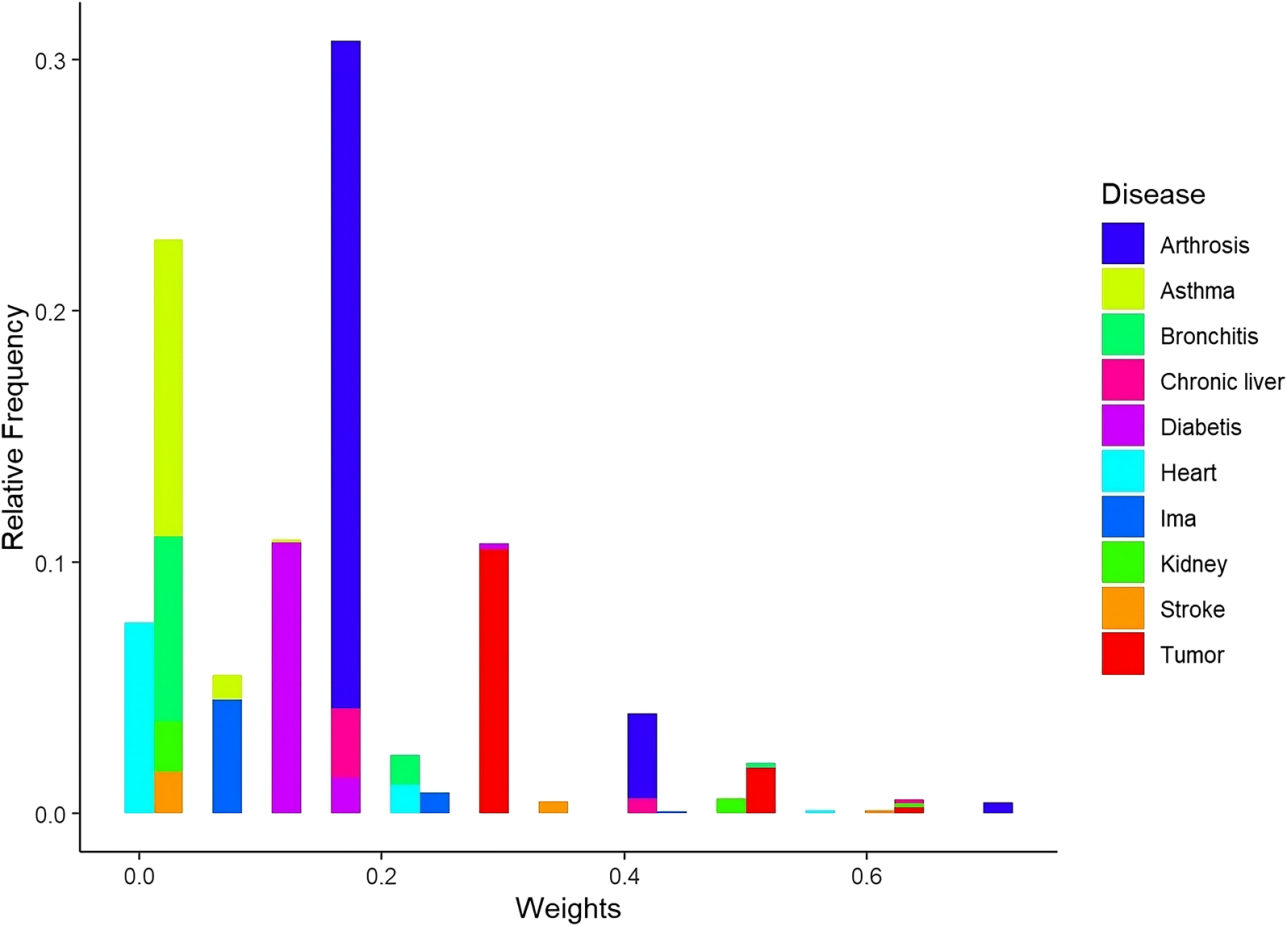
Relative frequencies of the filtered disability weights (i.e., after the text mining step) for each detected disease from the Italian surveillance system PASSI, considering the year 2019 with $n= 31,746$ respondents

As a first step, we selected the diseases considered by the GBD 2019 study that recall the diseases detected by PASSI. For example, focusing on diabetes, we selected through a text mining process all those weights that refer to diseases containing the words “diabet,” “diabetes,” “diabetic,” “diabeetus,” “diabetes mellitus,” “hypertension,” “obesity,” and “insulin.” We deal with singular and plural, and the keywords include synonymous terms from the Cambridge English dictionary [30]. The complete list of keywords used for each disease is reported in Table 2, while the corresponding selected disability weights in Table 3 in Appendix 2. Looking at Table 3, someone might opine that the proposed method also considers very rare health states by not adequately describing the analyzed population. However, in pursuit of a comprehensive and versatile approach for potential application in diverse contexts, we opted to incorporate all health states. Researchers interested in comparing results with or without considering rare diseases within the multi-step index construction process may omit these health states manually from the list in Table 3. Instead, Fig. 1 shows the relative frequencies of the filtered disability weights (i.e., after the text mining step explained before) for each detected disease from PASSI. From Fig. 1, we can note high disability weights are associated with individuals affected by tumors. At the same time, arthrosis has more variability (i.e., standard deviation equals 0.233), which will be handled in the second step.

**Fig. 2:**
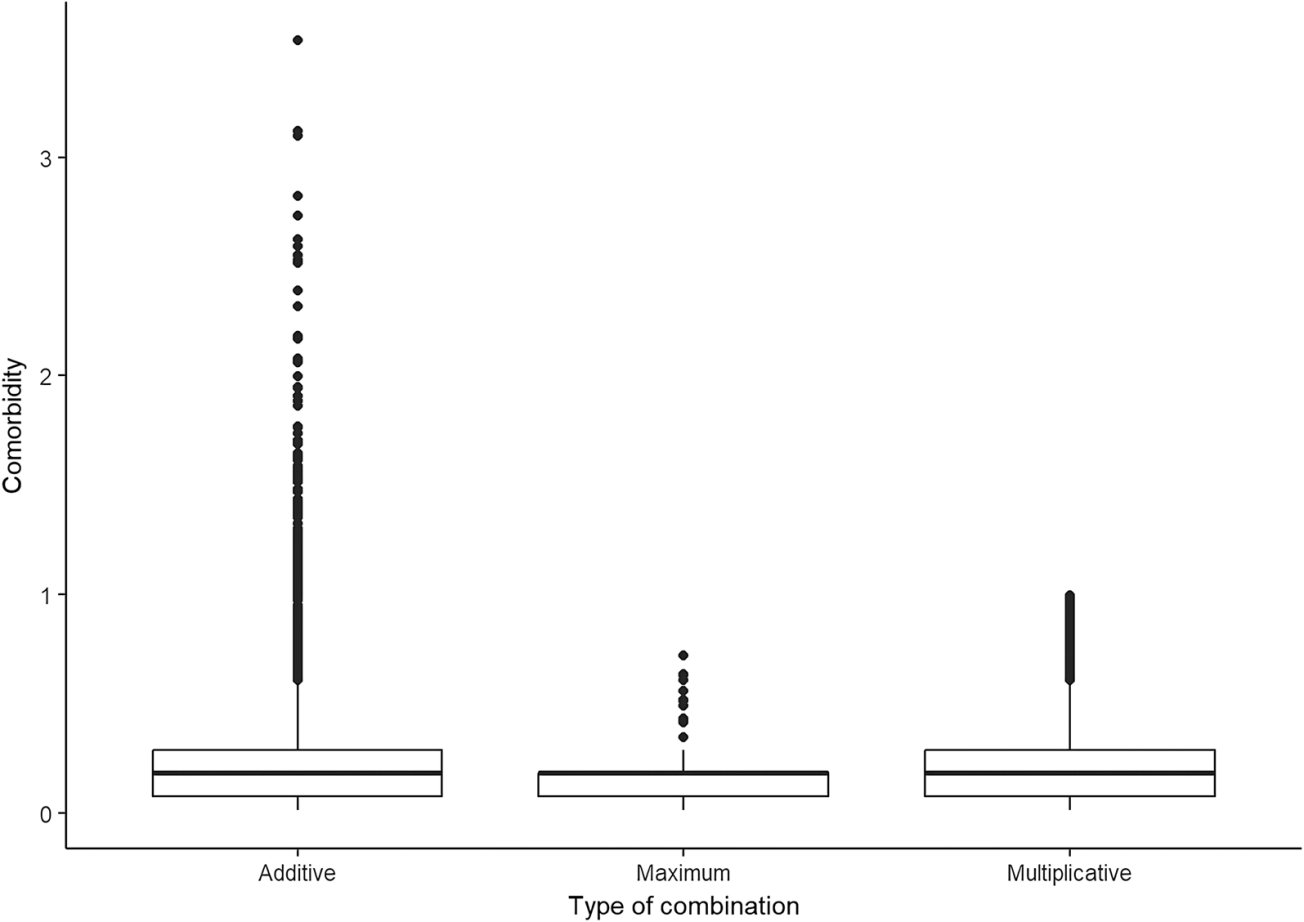
Boxplots of the comorbidity indexes based on the PASSI data (related to the 2019 year with $n= 31,746$ respondents) and GBD weights. The left boxplot corresponds to the distribution of the combined disability weights using Eq. (1) (i.e., additive approach), the center boxplot refers to the combination defined in Eq. (2) (i.e., maximum approach), and finally, the right boxplot corresponds to the comorbidity index created using Equation (3) (i.e., multiplicative approach)

**Fig. 3:**
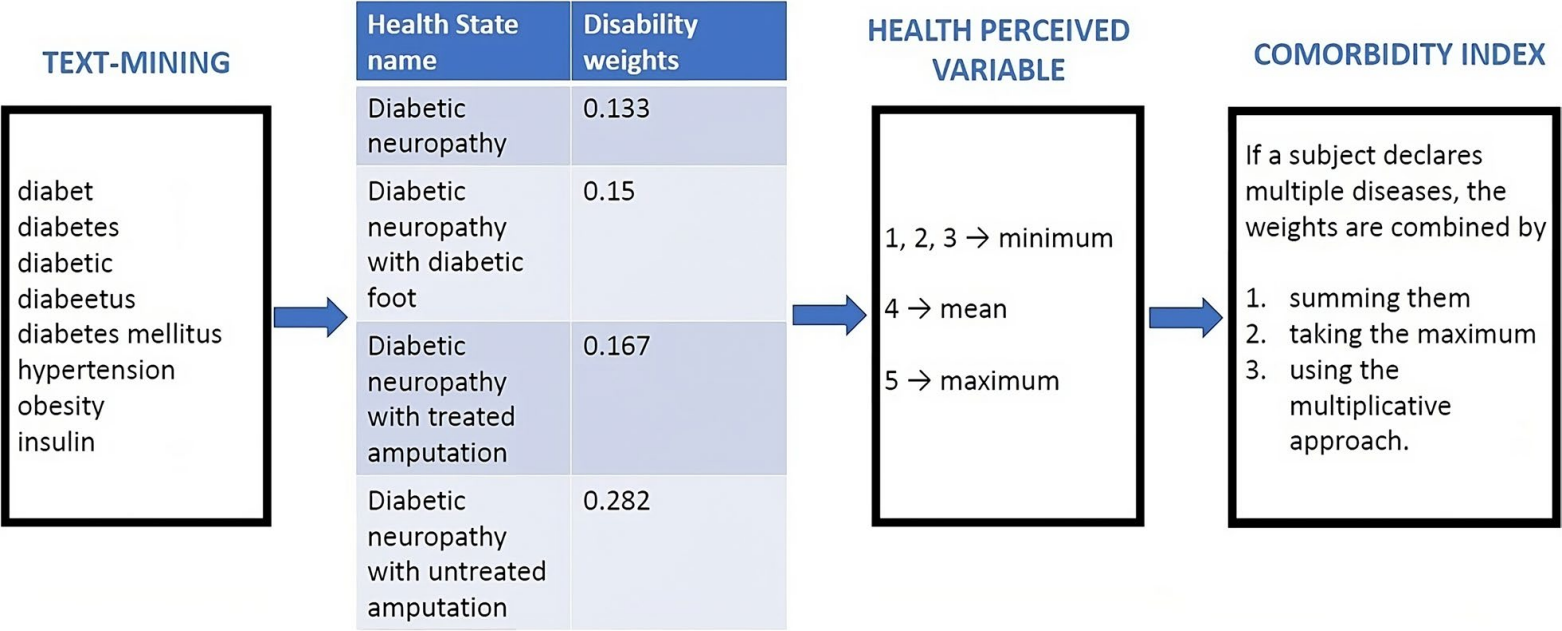
Steps to associate the weights coming from the GBD to the NCDs declared in the Italian surveillance system PASSI

**Fig. 4:**
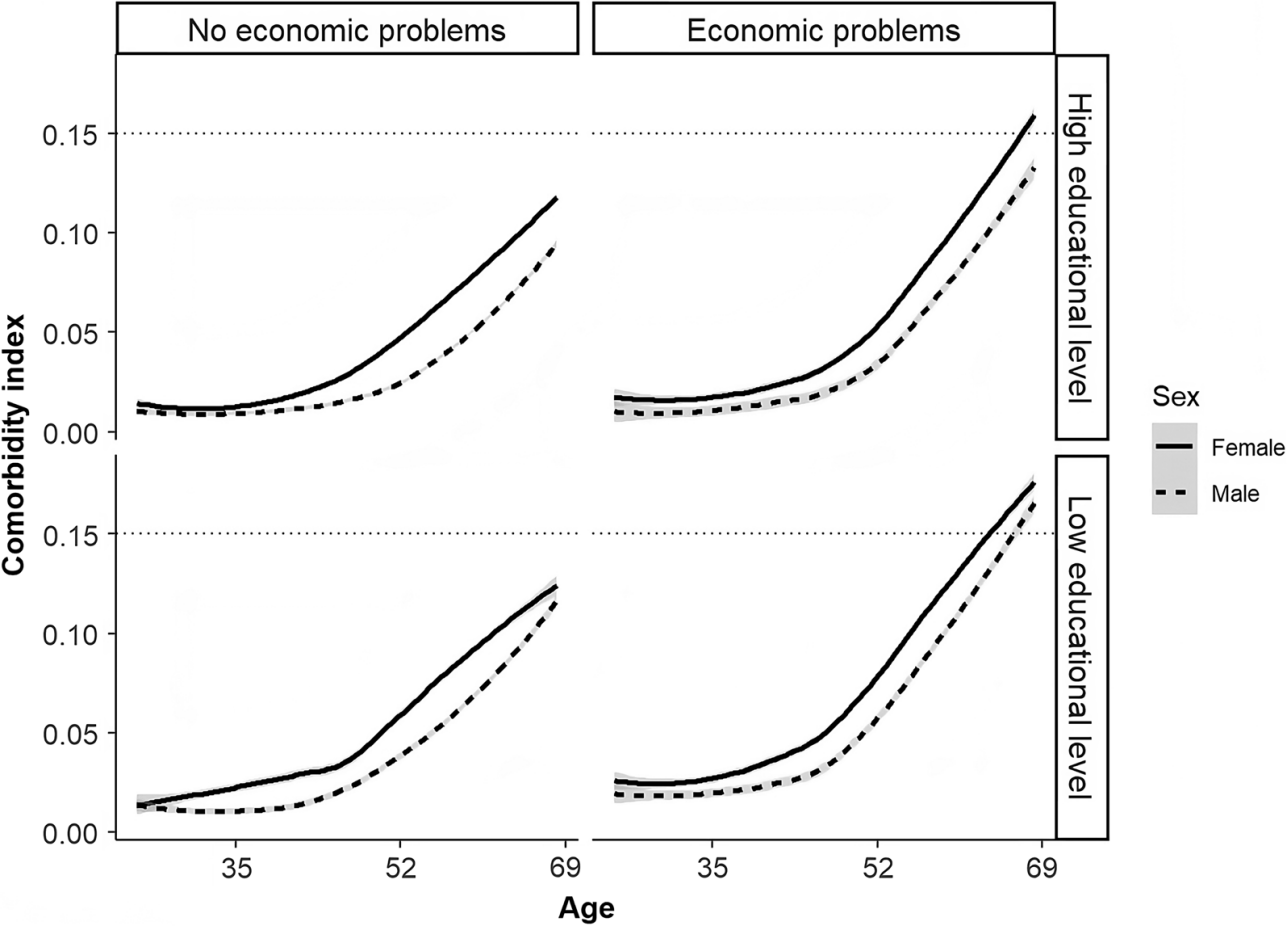
Predictions of the comorbidity index across age considering sub-populations characterized by different levels of education (low-high) and economic status (no economic problems-economic problems) in 2019. The gray area represents the prediction interval at level 0.95. The educational variable takes the value as “low” if the respondent has an educational level below high school and “high” otherwise. The economic variable assumes the level “no economic problem” if the respondent makes ends meet with the financial resources available (from own or family income) very/quite easily and “economic problem” otherwise

**Fig. 5:**
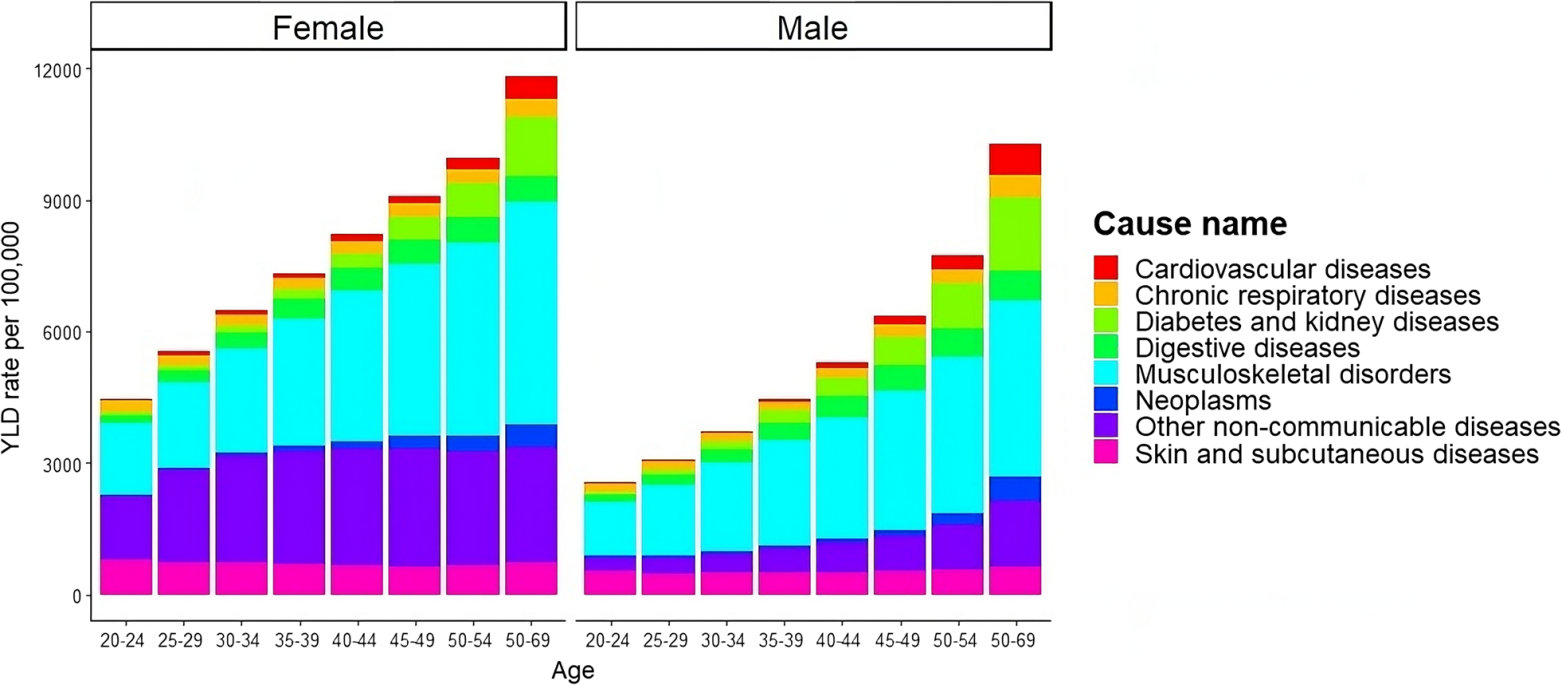
Years Lived with Disability (YLD) rate per 100, 000 population across age divided by sex using the GBD estimates (http://ihmeuw.org/5z0s, http://ihmeuw.org/5z0t) focusing on the 2019 year and NCDs related to the ones detected from the Italian surveillance system PASSI

From the first step, for example, still focusing on diabetes, we found 4 weights with a standard deviation equal to 0.06. To choose which of these 4 weights to associate with the individual who declared having diabetes in PASSI, we use the perceived health variable detected in PASSI described in the previous subsection. Thus, if perceived health is between 1 and 3, we use the minimum value of the weights. If it equals 4, we use the average of the selected disability weights, and if it equals 5, we consider the maximum value of these weights. So, here we are assuming that if a subject declared very bad health and more than one disease, both diseases strongly impact the subject’s life.

The final step is to account for multimorbidity. It is well known that ignoring the presence of more than one health condition in the estimation of disease burden measures leads to inaccurate results and conclusions, particularly if the elderly population is analyzed [18]. Therefore, if an individual declares more than one disease, we combine the selected disability weights by three types of combination functions described in the following.

Let us define as $W_{ij}$, where $i, \dots , 10$ and $j = 1, \ldots , n$ with *n* is the total number of subjects interviewed, the disability weight associated with the detected disease *i* in PASSI for subject *j*. We consider $W_{ij}=0$ if subject *j* does not declare the disease *i*. If subject *j* declares more than one disease, that is, $|\{i \in \{1, \dots , 10\}: W_{ij} \ne 0\}| > 1$ where $|\cdot |$ stands for the cardinality of the set, we combine $\{W_{ij}\}$ following the approaches proposed by Hilderink and colleagues [31] to create a combined disability weight $D_j = f(W_{1j}, \dots , W_{10j})$ for each subject *j* as following:

1 $$\begin{aligned} D_j^{\text {sum}}&= \sum _{i=1}^{10} W_{ij} \end{aligned}$$

2 $$\begin{aligned} D_j^{\text {max}}&= \max _{i=1, \dots , 10} W_{ij} \end{aligned}$$

3 $$\begin{aligned} D_j^{\text {mult}}&= 1- \prod _{i=1}^{10} (1- W_{ij}) . \end{aligned}$$

We will call the first approach, i.e., Equation (1) as “additive,” the second one, i.e., Equation (2) as “maximum,” and the last one, i.e., Equation (3) as “multiplicative.” Figure 2 shows the distribution of the proposed comorbidity indexes considering the three types of combinations, that is, Equations (1), (2), and (3).

We considered these combination functions mainly for two reasons: (i) they are the most widely used and validated in the literature [31–34], and (ii) the interpretation of the final combinations is quite simple. In the literature, some empirical studies prefer the multiplicative approach [32], which is also used by GBD to combine the disability weights in the 2010, and 2013’s analysis [35, 36], while others prefer the maximum one [33, 34]. However, we will apply all of them in our analysis to understand if the results are consistent beyond the final type of combinations used to construct the comorbidity index since each has pros and cons.

For example, the additive approach is not bounded from 0 to 1, which is a desirable property, since we construct the comorbidity index from weight values, unlike the last two methods. In addition, each approach makes some assumptions: the maximum approach considers only the most impactful disease within the life of the individual who declared more than one disease; the additive approach assumes a constant disability increment associated with a particular single health condition or with the presence of other ones. Finally, the multiplicative approach assumes that the proportion of increment in disability associated with a specific health condition is constant in any context, either in isolation as a single disease or with the presence of other health states [31, 37], i.e., each additional health condition increases functional disability relative to its previous level [32, 34]. Figure 3 summarizes this multi-step procedure considering diabetes as an example for the first step (i.e., text mining one).

We emphasize here that the approach’s simplicity renders it adaptable to diverse contexts. For example, it can be employed with data from the US Behavioral Risk Factor Surveillance System (BRFSS) that uses a questionnaire similar to that of PASSI. Researchers interested in its application elsewhere can utilize the disability weights available here https://ghdx.healthdata.org/record/ihme-data/gbd-2019-disability-weights and customize their list of keywords. Alternatively, they can use the list suggested in Appendix 1 if examining the same diseases of the paper. In this way, risk factors measured by the questionnaires, such as social, economic, or lifestyle situations, can be analyzed. In fact, this information is not generally available in administrative and hospital data and has not been investigated by GBD studies.

Lastly, it is important to highlight that incorporating disability weights in the index construction, as an alternative to directly analyzing the perceived health variable alone, enables the assessment of diverse illnesses’ varying impacts on an individual. Different diseases have different degrees of burden, due to the disability they imply, the sequelae, and the age at onset. In our case, the diseases considered are chronic; thus, duration is not an issue. The combination of diseases introduces more complexity in calculating disability and burden. However, calculating the comorbidity burden is crucial to understanding its implications in society and allows stratification by socioeconomic variables. For example, if an individual reports feeling severely ill but has conditions that have a minor impact on their quality of life, applying the disability weights will moderate the effect, concentrating primarily on lower values. This adjustment, although considering the highest value within the multi-step procedure, ensures a more reliable representation regarding the burden of the diseases on the subject’s quality of life since the GBD disability weights were validated many times in the literature [24, 26].

## Results

This section proposes a naive utilization of the comorbidity index defined in previous section. Looking at Figs. 1, 2, the response variable we want to analyze has a particular distribution. The comorbidity index appears to be a “semi-continuous” multimodal skewed nonnegative variable with several zero values. We then decide to use nonparametric methods, such as machine learning methods, that can handle any functional form of the analyzed response variable [38]. Here, we report the results coming from the random forest approach [27]. However, other methods can be used and compared (e.g., Tweedie regression [39], support vector machine [40]), but it is beyond the scope of this paper. In brief, random forest regression is a machine-learning method that forms a collection of decision trees. Each tree, structured with nodes representing decisions or tests on data features and leaf nodes indicating outputs or predictions, operates independently to provide predictions. The collective outcomes of these trees are averaged to yield the final prediction. We apply the random forest method, considering all three combinations to construct the comorbidity index, and interestingly, we found similar results.

**Table 1:** Variable importance measures from the random forest model for each covariate inserted into the model (i.e., age, sex, educational level (low-high), and economic status (no economic problems-economic problems)) using 2019 PASSI data (i.e., $n= 31,746$ respondents)

Variable	Importance
Age	100.000
Educational level	10.718
Sex	6.148
Economic problems	0

First, the importance of the covariates analyzed to predict the level of comorbidity, i.e., age, sex, educational status, and economic problems, is the same (in terms of order) across the results from the three combinations. We then report only one in Table 1. The importance is calculated as follows: the method permutes the feature values of each variable and computes the out-of-bag error (mean squared error in this case). The importance score, defined by Strobl and colleagues [41], is then calculated by averaging the difference in the out-of-bag error before and after the permutation over all trees. If the prediction error changes consistently, the related variable is defined as important inside the random forest model. The permutation-based importance measures are then scaled to have a maximum equal to 100 and a minimum equal to 0. Finally, this importance score is conditional in the sense of coefficients in regression models considering both the main and interaction effects of the variable [41]. We can note that age is the main variable that impacts the split of the random forest trees, having an importance score equal to 100. In contrast, the economic problems variable has a minimal effect on the model’s results, i.e., the importance score equals 0. This is probably due to the presence of a strong association between the economic and educational level variables.

**Fig. 7:**
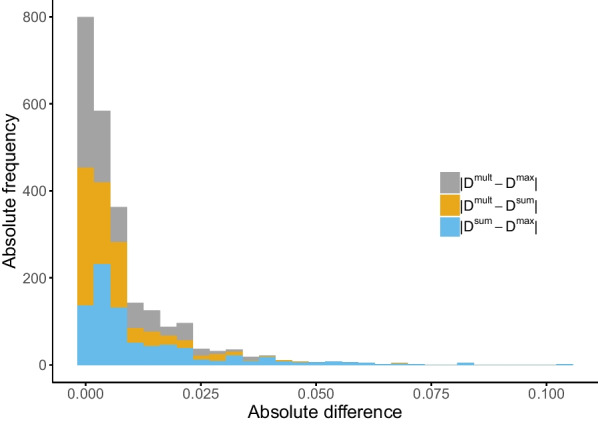
Histogram representing the distribution of the pairwise absolute differences between predictions (i.e., combined disability weights) using the three combination functions described in Equations (1), (2), and (3) (i.e., additive, maximum and multiplicative approaches)

**Fig. 8:**
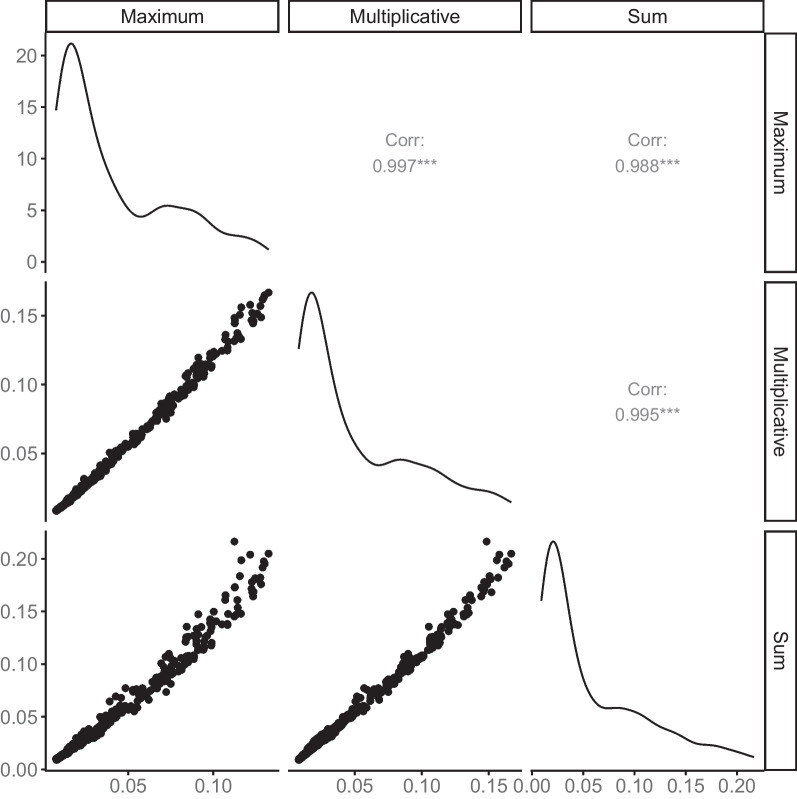
Pairwise scatterplots and density plots of the predictions in terms of combined disability weights using the three combination functions described in Equations (1), (2), and (3) (i.e., additive, maximum and multiplicative approaches)

Secondly, the trend of the predicted values across ages for each sub-population characterized by different sex, education, and economic status is very similar between the results from the three types of combinations. There is only a slightly greater separation between males and females in older ages with economic problems. However, the difference in terms of the mean absolute difference between predicted values using different approaches remains minimal, i.e., we have a mean absolute difference of 0.0058 (standard deviation equals 0.008) if the additive and multiplicative methods are considered, 0.0123 (standard deviation equals 0.015) if the comparison between additive and maximum is examined, and 0.0066 (standard deviation equals 0.008) if the last comparison is analyzed (i.e., between multiplicative and maximum approaches). Figures 7 and 8 in Appendix 3 show the absolute frequencies considering the absolute pairwise differences between these predictions and some exploratory plots to understand the relationship between them.

**Fig. 9:**
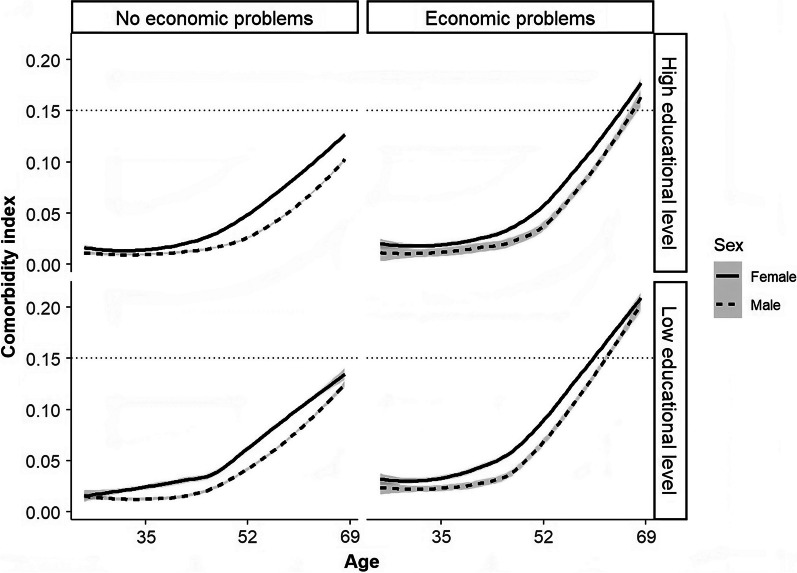
Predictions of the comorbidity index across age considering sub-populations characterized by different levels of education (low-high) and economic status (no economic problems-economic problems). The gray area represents the prediction interval at level 0.95. The comorbidity index refers to the one constructed using Equation (1) (i.e., additive approach) and 2019 PASSI data (i.e., $n= 31,746$ respondents). The educational variable takes the value as “low” if the respondent has an educational level below high school and “high” otherwise. The economic variable assumes the level “no economic problem” if the respondent makes ends meet with the financial resources available (from own or family income) very/quite easily and “economic problem” otherwise

**Fig. 10:**
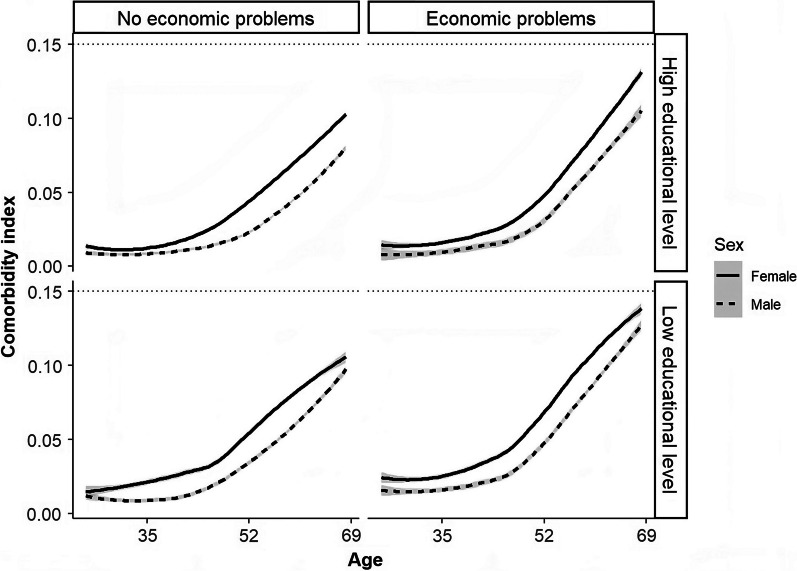
Predictions of the comorbidity index across age considering sub-populations characterized by different levels of education (low-high) and economic status (no economic problems-economic problems). The gray area represents the prediction interval at level 0.95. The comorbidity index refers to the one constructed using Equation (2) (i.e., maximum approach) and 2019 PASSI data (i.e., $n= 31,746$ respondents). The educational variable takes the value as “low” if the respondent has an educational level below high school and “high” otherwise. The economic variable assumes the level “no economic problem” if the respondent makes ends meet with the financial resources available (from own or family income) very/quite easily and “economic problem” otherwise

Therefore, we report here only the results considering the multiplicative approach being the one with a mean absolute difference lower for both comparisons, while the predictions using the additive approach (i.e., Equation (1)) and the maximum one (i.e., Equation (2)) are shown, respectively, in Figs. 9 and 10 in Appendix 3. Figure 4 shows the predicted values of the GBD disability weights across age, analyzing 4 populations characterized by different economic (no economic problem, economic problem) and educational (low-medium/high) status levels divided by sex. As expected, We can note how the disability weights increase as age increases. We can note a great difference between males and females, particularly in the elderly, if the sub-population characterized by a high educational level is considered (i.e., left and right top plots of Fig. 4).

More interestingly, in older ages, the comorbidity index is lower in the sub-population characterized by high educational level and no economic problems (i.e., left top plot of Fig. 4). For example, focusing on the elderly population (i.e., age equals 69), the comorbidity index equals 0.124 for the females and 0.088 for the males if the sub-population with a high educational level and no economic problems is analyzed. In contrast, it equals 0.164 for the females and 0.146 for the males if the sub-population with a low educational level and economic problems is considered (i.e., right bottom plot of Fig. 4). In addition, we can note how the difference in terms of comorbidity index is substantial also in adult ages, not only in elderly ages if the sub-population characterized by economic problems and low educational level is analyzed (e.g., the index equals 0.074 for females and 0.049 for males at age 49). According to the literature, these statements support the presence of a difference in terms of comorbidity in socioeconomic class [42, 43].

Finally, the predicted values reported in Fig. 4 are in line with the analysis of the Years Lived with Disability (YLD) index coming from the GBD 2019 if only the division by age and sex is considered, which are the only one available from the GBD project. Figure 5 shows the YLDs considering the same range of age of PASSI and diseases related to the ones detected in PASSI (i.e., diabetes and kidney diseases, cardiovascular diseases, neoplasms, digestive diseases, other non-communicable diseases, skin, and subcutaneous diseases, chronic respiratory diseases and musculoskeletal disorders). Therefore, thanks to the proposed new comorbidity burden index, we can also analyze the level of comorbidity of the Italian population characterized by different educational levels and economic status, which the GBD Project does not detect.

## Discussion

With populations aging, the study of comorbidity and disability and their dynamics is more and more relevant. Policymakers and decision-makers need timely information on the evolution of morbidity and comorbidity and their impact on disability, particularly when, typically, with aging, the prevalence of multiple chronic diseases increases. NCDs surveillance systems can offer timely information on the evolution of diseases together with other sociodemographic fundamental details. On the impact of diseases on disability, GBD has done globally precious work to estimate the impact of morbidity on disability, eventually becoming one of the most relevant measures for policies. In this paper, we proposed a new index that measures morbidity and comorbidity by analyzing the Italian surveillance system PASSI data and the disability weights given by the GBD 2019 study. The NCDs detected in PASSI were associated with the disability weights of the GBD through several steps: a text mining one to extract the related GBD weights and the utilization of the perceived health variable reported in PASSI to filter the extracted GBD weights.

We finally proposed a naive analysis of this comorbidity burden index considering sub-populations characterized by sex, age, and different levels of education and economic status of the subjects. Interestingly, we found minimal differences in predicted values of comorbidity if the additive, multiplicative, or maximum approaches were used to combine the disability weights. Comparing our results with the ones from previous studies on morbidity from the same surveillance data [19], we can underline mainly three differences: (i) Pastore and colleagues [19] found that females have a lower probability of having at least one disease than males in elderly ages, while we found greater levels of comorbidity burden index in females than males; (ii) the sex difference in older ages seems to equal between socioeconomic sub-populations in the work of Pastore and colleagues [19], while we found greater differences in underprivileged/privileged sub-population; (iii) the onset of comorbidity in disadvantaged sub-populations seems to start earlier using the comorbidity index proposed compared to the results found by Pastore et al. [19]. We argue these differences underlying the fact that Pastore and colleagues [19] analyzed morbidity, i.e., the presence of at least one disease, without considering the burden of these diseases on the people’s health quality and the co-presence of these diseases. Instead, we take the impact of disability into account (thanks to the incorporation of the disability weights), and finding a worse situation for some population’s subgroups is a sign that we are indeed analyzing the effect of comorbidity and its impact on health quality. In fact, our results align with those from the GBD in terms of years lived with disability (YLD).

Interestingly, it also shows how relevant health inequalities are when observing these morbidity-comorbidity indexes among sub-populations. If higher risk factors prevalence among more deprived groups has been globally proved, see other studies on PASSI data [42], as well as the prevalence of multiple risk factors [44], to show the relevance of these on morbidity and their impact on disability is an additional, relevant, information. Comparisons with other indexes proposed in the literature are difficult since these, so far, are based on data coming from sources too different from those used. This is why we have limited the comparison with GBD results and indexes utilized PASSI data (i.e., the work of Pastore et al. [19]).

Some limitations of the method are, however, worth emphasizing. The method for identifying comorbidities involves extracting texts using the synonyms of the diseases reported by the respondents in the PASSI questionnaire. As the health conditions are self-reported, information bias may affect the index proposed. Furthermore, using the perceived health variable to associate disability weights with the reported diseases introduces its challenges. Respondents could report their perceived health level also considering other factors (e.g., mental ones) besides the effects of their chronic diseases, leading to potential inaccuracies or variations in their responses. Despite these challenges, the method has been validated against data from the GBD study (i.e., analyzing the YLD index). Additionally, results based on the latent variable model support the findings reported in our paper. Notably, the perceived health variable was not used in the latent trait model. In addition, it must be emphasized that the keywords used in the text-mining step comprise synonyms for the diseases reported in PASSI. Therefore, a better selection of words with the help of experts in the field could be helpful. However, we have proceeded in this way to propose a simple, effective, and fast in terms of implementation comorbidity burden index that can also be used (with appropriate modifications if necessary) on data from other questionnaires (e.g., BRFSS [45]) and give an insight view of comorbidity in the Italian subpopulation characterized by different socioeconomic levels.

In conclusion, the comorbidity burden index proposed permits exploring two novel analyses: the level of comorbidity from surveillance systems, like PASSI, and the socioeconomic population structure of the GBD estimates in a simple way. As further directions, the list of keywords should be validated by experts. Analyzing the comorbidity trend would be interesting by comparing different years of the PASSI survey as previously done in the work of Pastore et al. [19] on a more general and gross morbidity indicator. Finally, utilizing the same approach could be of interest in studying international comparison applying to other NCDs’ surveillance data.

## Figures and Tables

**Fig. 6:**
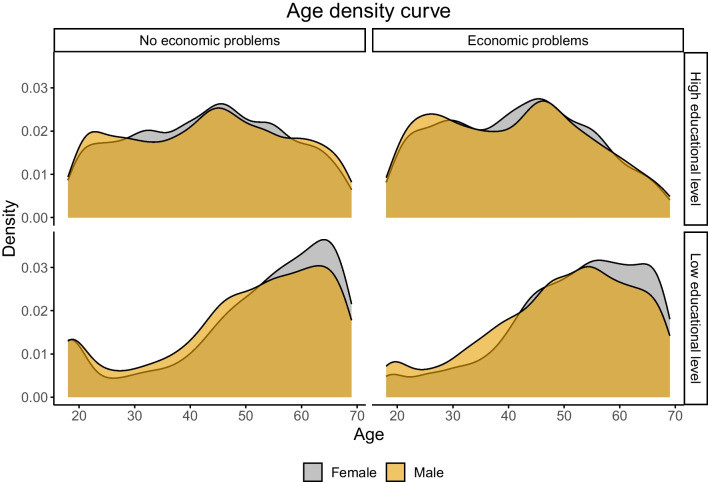
Density plots showing the distribution of the variable age divided by sex for each subpopulation characterized by different educational (low-high) and economic (no economic problems-economic problems) levels for the PASSI sample analyzed (i.e., year 2019 with $n= 31,746$ respondents). The educational variable takes the value as “low” if the respondent has an educational level below high school and “high” otherwise. The economic variable assumes the level “no economic problem” if the respondent makes ends meet with the financial resources available (from own or family income) very/quite easily and “economic problem” otherwise

## Data Availability

The data set containing the disability weights from the GBD project is available at https://ghdx.healthdata.org/record/ihme-data/gbd-2019-disability-weights. The data set from the surveillance system PASSI is available from the authors upon reasonable request.

## Appendix 1: PASSI data

Figure 6 shows the density distribution of the variable age for the PASSI sample divided by sex, educational (low-high), and economic (no economic problems-economic problems) levels, focusing on 2019 year data.

## Appendix 2: Keywords diseases list and GBD disability weights

Table 2 reports the keywords used in the first step for each disease detected from the Italian surveillance system PASSI. Table 3 shows the selected disability weights after the first step described in Fig. 3 using the keywords defined in Table 2 for each disease detected in PASSI.

**Table 2:** List of keywords for each detected disease in the Italian surveillance system PASSI used in the first step of Fig. 3

Disease	Keywords
Kidney failure	renal, kidney, nephritic, intrarenal, nephrotic, nephric, bladder
Bronchitis, emphysema, respiratory failure	bronchitis, pneumonia, sinusitis, emphysema, rhinitis, COPD
Myocardial infarction, cardiac ischemia, coronary artery disease	myocardium, cardiac, coronary, artery, ischemia, ischaemia, ischemic, cardio, cardiovascular, cardial, myocardial, coronal
Tumor (including leukemias and lymphomas)	tumors, tumos, cancers, cancer, carcinomia, neoplasm, malignancy, leukemia, lymphoma, blood, lymphomas, lymphoma, hogkin, myeloma, sarcoma, B-cell, osteosarcoma, myelodysplasia
Chronic liver disease, cirrhosis	liver, hepatic, pancreas, stomach sweetbread, cirrhosis, cirrhotic, chronic, fibrosis, pancreatitis, liverwort, biliary
Stroke or cerebral ischemia	stroke, apoplexy, cerebrovascular, cva, ischemia, ischaemia, vasospasm, hypoxia,reperfusion, anoxia, infarction, dysrhythmia, infarct, hypoperfusion, myocardium, hypoxia, infarction, ischemic
Other heart diseases (e.g., valvulopathy decompensation)	heart, valvulopathy, valvular, valve, valvulopathies, valves
Bronchial asthma	asthma, bronchial, bronchitis, suffocative catarrh, allergy
Arthrosis or Arthritis (e.g., rheumatoid arthritis, gout, lupus, fibromyalgia)	arthrosis, arthritis, Osteoarthritis, Arthroses, ankylosis, osteoarthrosis, arthropathy, osteochondrosis, osteophyte, rheumatoid, rheumatoid, gout, lupus, fibromyalgia, sclerosis, polyarthritis

**Table 3:** GBD weights after the first step defined in Fig. 3 (i.e., text-mining one). The first column represents the diseases detected in PASSI, the second one the health state name of the GBD disability weights, and the last one the corresponding disability weights

**Disease (PASSI)**	**Health state name (GDB)**	**Disability weight**
Diabetis	Diabetic neuropathy	0.133
Diabetis	Diabetic neuropathy with diabetic foot	0.150
Diabetis	Diabetic neuropathy with treated amputation	0.167
Diabetis	Diabetic neuropathy with untreated amputation	0.282
Kidney failure	Terminal phase, with medication (for cancers, end-stage kidney/liver disease)	0.540
Kidney failure	Terminal phase, without medication (for cancers, end-stage kidney/liver disease)	0.569
Kidney failure	End-stage renal disease, with kidney transplant	0.024
Kidney failure	End-stage renal disease, on dialysis	0.571
Kidney failure	End-stage renal disease, on dialysis and mild anemia	0.573
Kidney failure	End-stage renal disease, on dialysis and moderate anemia	0.593
Kidney failure	End-stage renal disease, on dialysis and severe anemia	0.633
Kidney failure	Chronic kidney disease (stage IV)	0.104
Kidney failure	Mild anemia and terminal phase, without medication (for cancers, end-stage kidney/liver disease)	0.570
Kidney failure	Moderate anemia and terminal phase, without medication (for cancers, end-stage kidney/liver disease)	0.591
Kidney failure	Severe anemia and terminal phase, without medication (for cancers, end-stage kidney/liver disease)	0.631
Bronchitis	COPD and other CR problems, mild	0.019
Bronchitis	COPD and other CR problems, moderate	0.225
Bronchitis	COPD and other CR problems, severe	0.408
Bronchitis	Severe COPD and other CR, with mild heart failure	0.432
Bronchitis	Severe COPD and other CR, with moderate heart failure	0.450
Bronchitis	Severe COPD and other CR, with severe heart failure	0.512
Bronchitis	COPD and other CR problems, severe and generic uncomplicated disease: worry and daily medication	0.437
Bronchitis	Mild abdominopelvic problem and mild COPD and other CR problems	0.030
Bronchitis	Level 1 disfigurement with itch/pain and mild COPD and other CR problems	0.045
Bronchitis	Mild intellectual disability and mild COPD and other CR problem	0.061
Bronchitis	Mild abdominopelvic problem, mild COPD and other CR problems, and level 1 disfigurement with itch/pain	0.056
Bronchitis	Mild COPD and other CR problems, mild intellectual disability, and level 1 disfigurement with itch/pain	0.086
Bronchitis	Mild abdominopelvic problem, mild COPD and other CR problems, mild intellectual disability, and level 1 disfigurement with itch/pain	0.096
Bronchitis	Mild abdominopelvic problem, moderate abdominopelvic problem, and mild COPD and other CR problems	0.141
Myocardial infarction	Cardiac conduction disorders and cardiac dysrhythmias	0.224
Myocardial infarction	Acute myocardial infarction, days 1-2	0.432
Myocardial infarction	Acute myocardial infarction, days 3-28	0.074
Tumor	Cancer, diagnosis and primary therapy	0.288
Tumor	Cancer, metastatic	0.451
Tumor	Terminal phase, with medication (for cancers, end-stage kidney/liver disease)	0.540
Tumor	Terminal phase, without medication (for cancers, end-stage kidney/liver disease)	0.569
Tumor	Mild anemia and terminal phase, without medication (for cancers, end-stage kidney/liver disease)	0.570
Tumor	Moderate anemia and terminal phase, without medication (for cancers, end-stage kidney/liver disease)	0.591
Tumor	Severe anemia and terminal phase, without medication (for cancers, end-stage kidney/liver disease)	0.631
Liver	Terminal phase, with medication (for cancers, end-stage kidney/liver disease)	0.540
Liver	Terminal phase, without medication (for cancers, end-stage kidney/liver disease)	0.569
Liver	Decompensated cirrhosis of the liver	0.178
Liver	Decompensated cirrhosis of the liver and mild anemia	0.181
Liver	Decompensated cirrhosis of the liver and moderate anemia	0.220
Liver	Decompensated cirrhosis of the liver and severe anemia	0.300
Liver	Mild anemia and terminal phase, without medication (for cancers, end-stage kidney/liver disease)	0.570
Liver	Moderate anemia and terminal phase, without medication (for cancers, end-stage kidney/liver disease)	0.591
Liver	Severe anemia and terminal phase, without medication (for cancers, end-stage kidney/liver disease)	0.631
Stroke	Cardiac conduction disorders and cardiac dysrhythmias	0.224
Stroke	Acute myocardial infarction, days 1-2	0.432
Stroke	Acute myocardial infarction, days 3-28	0.074
Stroke	Stroke, LT consequences, mild	0.019
Stroke	Stroke, LT consequences, moderate	0.070
Stroke	Stroke, LT consequences, moderate plus cognition problems	0.316
Stroke	Stroke, LT consequences, severe	0.552
Stroke	Stroke, LT consequences, severe plus cognition problems	0.588
Stroke	Generic uncomplicated disease anxiety; LT consequences due to stroke	0.325
Stroke	Generic uncomplicated disease anxiety; LT consequences due to stroke severe abdominopelvic problem	0.541
Stroke	Generic uncomplicated disease anxiety; LT consequences due to stroke; Anemia severe	0.424
Stroke	Generic uncomplicated disease anxiety; LT consequences due to stroke; Anemia severe; severe abdominopelvic problem	0.607
Heart	Heart failure, mild	0.041
Heart	Heart failure, moderate	0.072
Heart	Heart failure, severe	0.179
Heart	Severe COPD and other CR, with mild heart failure	0.432
Heart	Severe COPD and other CR, with moderate heart failure	0.450
Heart	Severe COPD and other CR, with severe heart failure	0.512
Heart	Congenital heart disease	0.061
Heart	Congenital heart disease and mild heart failure	0.041
Heart	Congenital heart disease and moderate heart failure	0.072
Heart	Congenital heart disease and severe heart failure	0.179
Heart	Congenital heart disease, borderline intellectual functioning, and mild heart failure	0.052
Heart	Congenital heart disease, borderline intellectual functioning, and moderate heart failure	0.082
Heart	Congenital heart disease, borderline intellectual functioning, and severe heart failure	0.188
Heart	Congenital heart disease, mild intellectual disability, and mild heart failure	0.082
Heart	Congenital heart disease, mild intellectual disability, and moderate heart failure	0.111
Heart	Congenital heart disease, mild intellectual disability, and severe heart failure	0.214
Heart	Congenital heart disease, moderate intellectual disability, and mild heart failure	0.137
Heart	Congenital heart disease, moderate intellectual disability, and moderate heart failure	0.164
Heart	Congenital heart disease, moderate intellectual disability, and severe heart failure	0.261
Heart	Congenital heart disease, severe intellectual disability, and mild heart failure	0.195
Heart	Congenital heart disease, severe intellectual disability, and moderate heart failure	0.220
Heart	Congenital heart disease, severe intellectual disability, and severe heart failure	0.310
Heart	Congenital heart disease, profound intellectual disability, and mild heart failure	0.233
Heart	Congenital heart disease, profound intellectual disability, and moderate heart failure	0.257
Heart	Congenital heart disease, profound intellectual disability, and severe heart failure	0.342
Heart	Congenital heart disease and borderline intellectual functioning	0.011
Heart	Congenital heart disease and Intellectual disability/ mental retardation, mild	0.043
Heart	Congenital heart disease and Intellectual disability/ mental retardation, moderate	0.100
Heart	Congenital heart disease and Intellectual disability/ mental retardation, severe	0.160
Heart	Congenital heart disease and Intellectual disability/ mental retardation, profound	0.200
Heart	Congenital heart disease and generic uncomplicated disease: worry and daily medication	0.049
Heart	Congenital heart disease and borderline intellectual functioning and generic uncomplicated disease:worry and daily medication	0.059
Heart	Congenital heart disease and Intellectual disability/ mental retardation, mild and generic uncomplicated disease: worry and daily medication	0.089
Heart	Congenital heart disease and Intellectual disability/ mental retardation, moderate and generic uncomplicated disease: worry and daily medication	0.144
Heart	Congenital heart disease and Intellectual disability/ mental retardation, severe and generic uncomplicated disease: worry and daily medication	0.201
Heart	Congenital heart disease and Intellectual disability/ mental retardation, profound and generic uncomplicated disease: worry and daily medication	0.239
Heart	Borderline intellectual disability with congenital heart disease	0.011
Heart	Mild intellectual disability with congenital heart disease	0.043
Heart	Moderate intellectual disability with congenital heart disease	0.100
Heart	Severe intellectual disability with congenital heart disease	0.160
Heart	Profound intellectual disability with congenital heart disease	0.200
Heart	Congenital heart disease and mild dementia	0.069
Heart	Congenital heart disease and moderate dementia	0.377
Heart	Congenital heart disease and severe dementia	0.449
Heart	Congenital heart disease, mild dementia, borderline intellectual disability	0.079
Heart	Congenital heart disease, mild dementia, mild intellectual disability	0.109
Heart	Congenital heart disease, mild dementia, moderate intellectual disability	0.162
Heart	Congenital heart disease, mild dementia, severe intellectual disability	0.218
Heart	Congenital heart disease, mild dementia, profound intellectual disability	0.255
Heart	Congenital heart disease, moderate dementia, borderline intellectual disability	0.384
Heart	Congenital heart disease, moderate dementia, mild intellectual disability	0.403
Heart	Congenital heart disease, moderate dementia, moderate intellectual disability	0.438
Heart	Congenital heart disease, moderate dementia, severe intellectual disability	0.475
Heart	Congenital heart disease, moderate dementia, profound intellectual disability	0.499
Heart	Congenital heart disease, severe dementia, borderline intellectual disability	0.455
Heart	Congenital heart disease, severe dementia, mild intellectual disability	0.472
Heart	Congenital heart disease, severe dementia, moderate intellectual disability	0.503
Heart	Congenital heart disease, severe dementia, severe intellectual disability	0.535
Heart	Congenital heart disease, severe dementia, profound intellectual disability	0.557
Heart	Congenital heart disease with primary infertility	0.068
Heart	Severe motor plus cognitive impairments and congenital heart disease	0.542
Bronchial asthma	Asthma, controlled	0.015
Bronchial asthma	Asthma, partially controlled	0.036
Bronchial asthma	Asthma, uncontrolled	0.133
Arthrosis	Multiple sclerosis, mild	0.183
Arthrosis	Multiple sclerosis, moderate	0.463
Arthrosis	Multiple sclerosis, severe	0.719
Arthrosis	Gout, acute	0.295

## Appendix 3: Comorbidity burden index predictions

Figure 7 shows the distribution of the pairwise absolute differences of the predictions in terms of disability weights combined by Equations (1), (2), and (3) (i.e., additive, maximum and multiplicative approaches).

Figure 8 represents the relationship between the predictions using the three different combination approaches defined in Equations (1), (2), and (3) (i.e., additive, maximum and multiplicative approaches).

Figure 9 shows the predictions of the comorbidity index across age considering sub-populations characterized by different levels of education (low-high) and economic status (no economic problems-economic problems) when the comorbidity index is constructed using the sum of disability weights (i.e., Equation (1)).

Figure 10 shows the predictions of the comorbidity index across age considering sub-populations characterized by different levels of education (low-high) and economic status (no economic problems-economic problems) when the comorbidity index is constructed using the maximum of disability weights (i.e., Equation (2)).

## Abbreviations

GBD: Global Burden of Disease
PASSI: Progressi delle Aziende Sanitarie per la Salute in Italia
NCDs: Non-Communicable Diseases
CR: Chronic Respiratory
LT: Long-Term
YLD: Years Lived with Disability
BRFSS: Behavioral Risk Factor Surveillance System
